# The British object and action naming test for intraoperative mapping (BOATIM): A standardised and clinically tested framework for awake brain surgery

**DOI:** 10.1007/s00701-025-06521-8

**Published:** 2025-04-15

**Authors:** Hajira Mumtaz, Anna E. Piasecki, Minna Kirjavainen, Margaret Newson, Madeleine Farrow, Molly Cree, Neil U. Barua

**Affiliations:** 1 Southmead Hospital, North Bristol NHS Trust, Bristol, UK; 2 Brain, Language, and Behaviour Laboratory; Centre for Health and Clinical Research, University of the West of England, Bristol, UK; 3 College of Arts, Technology, and Environment, University of the West of England, Bristol, UK

**Keywords:** Awake craniotomy, Language mapping, Intraoperative, Pre-operative, Post-operative, Picture naming

## Abstract

**Background:**

Picture-naming tasks are widely used for identifying speech-eloquent regions during awake craniotomy. However, language-specific and culturally relevant task stimuli remain scarce. Current practices mostly rely on translated stimuli that do not reflect the everyday language use of the target speakers and might be susceptible to misinterpretations due to linguistic and cultural differences. Additionally, non-standardised homemade tasks are used. Here, we, for the first time, present the development, standardisation, and clinical application of two tests designed specifically for functional mapping in British English.

**Methods:**

115 object and 86 action stimuli were developed using the British National Corpus (BNC) and controlled for confounding psycholinguistic variables using normative data from native speakers. Optimization of the items for intraoperative use was done by first standardising the tests in healthy volunteers followed by their application during the electrical stimulation of language-eloquent regions in brain tumour patients. In the standardised data, the influence of word- and subject-related factors on performance, and the test-retest reliability was explored.

**Results:**

The final items achieved above 80% naming agreement. Object naming proved easier compared to action naming, with accuracy positively influenced by word frequency and negatively affected by the age-of-acquisition variable in both tasks. No subject-related effects were found. Excellent test-retest reliability confirmed the consistency of the tests in measuring language abilities. Positive maps obtained during intraoperative functional mapping demonstrated the sensitivity of the tests in detecting speech-eloquent regions.

**Conclusion:**

The tests provide a reliable and robust tool containing stimuli that are linguistically and culturally appropriate to British-English speakers.

**Supplementary Information:**

The online version contains supplementary material available at 10.1007/s00701-025-06521-8.

## Introduction

In awake craniotomy, the detection of the boundaries of language-eloquent brain regions through intraoperative direct electrical stimulation (DES) is crucial not only for maximizing tumour resection and improving patient survival rates, but also for preventing permanent neurological deficits [15]. Picture-naming tasks, which prompt patients to name visual stimuli, are among the most widely used neuropsychological assessment tools that facilitate this identification process – commonly known as “intraoperative language mapping” – as they are sensitive to the 4-second time-constraint imposed by DES [30]. Additionally, they allow assessment of important language domains [37]. For instance, an object naming task is targeted at evaluating word access and retrieval, and the production of lexical-semantic and grammatical information (e.g., number and gender encoding) [30]. Action naming, while engaging similar functions, can further measure syntactic levels of language (e.g., syntactic operations required to establish subject-verb agreement relations, time reference) [31].

Naming tasks are also employed pre- and post-operatively as a part of patients’ broader neuropsychological evaluation. Pre-operatively, their administration determines patients’ eligibility for language mapping and identifies task items that are suitable to be used during surgery (i.e., these comprise items that the patient can name without any errors) [3] [20]. Post-operatively, patients’ neurocognitive status can be monitored to assess whether they require any language rehabilitation [30].

Despite the extensive use of these tasks in the neurosurgical setting, language-specific and culturally-tailored stimuli are scarce [37]. Existing assessments mostly rely on translations or adapted versions of tests originally developed in other languages [9] [35]. Such approaches are suboptimal as they overlook the linguistic and cultural diversity between normative samples, thereby resulting in stimuli that are biased and not representative of the everyday language use of the target speakers [27] [34]. Additionally, psycholinguistic variables such as word frequency, familiarity, and age-of-acquisition – that are known to impact naming accuracy and latency – vary across languages and cultural contexts: for example a concept may be more frequently used or familiar in one language/culture but not in another [37]. Given this correlation, task performance on translated stimuli can be influenced by cultural and linguistic differences. This has been previously evidenced by picture norming studies where discrepancies in naming behaviour appeared due to misinterpretations of the culturally unsuitable stimuli: for instance, British speakers were found to use variable names for less frequent (American) concepts like “baseball” [2]. Similarly French speakers from Canada showed poorer naming agreements compared to French speakers from France on the same picture stimuli [33]. To avoid such potential confounds in high-stake scenarios like neurosurgical evaluation, where highly reliably naming performance is a pre-requisite of successful testing, language-specific and culturally-relevant task stimuli are ideal [9].

A tradition of homemade tasks also exists in clinical practice [27]. The stimuli of these tasks, however, are not controlled for relevant psycholinguistic variables or pretested in healthy volunteers [31]. Results obtained from such assessments are, therefore, difficult to interpret or compare with previous or later evaluations [30]. Aside from this, items borrowed from well-known standardised batteries such as the Denomination Orale (DO80) [19] and Aachen Aphasia Test (AAT) [38] are also used [14] [15]. Such batteries, however, were distinctively developed for other pathologies (e.g., post-stroke aphasia) and are hence not capable of coping with DES settings [27].

In the current work, we set out to develop and standardise two tests for language mapping that are specifically addressed to British-English speakers: *The British object and action naming test for intraoperative mapping (BOATIM*). To the best of our knowledge, no standardised intraoperative test yet exist in British-English. Paradigms that have been previously proposed for use during DES for English language in general (i.e., VAN-POP [26] and MULTIMAP [12]) are both translations of the original Dutch and Spanish tests, respectively. The stimuli of these tests, being developed in a different cultural context, are biased and are not entirely suitable to British-English speakers. Importantly also, the clinical use of these tasks is very limited (so far only VAN-POP has been tested in three native English speakers [17]). A recent survey conducted by our research team [18] explored language mapping methods across the UK. The findings revealed that the most prevalent practice is the use of homemade tasks where stimuli are designed either within the institutions or translated from the Dutch Linguistic Intraoperative Protocol (DuLIP) battery [10].

With BOATIM, we present two sets of linguistically controlled stimuli (one for object naming and one for action naming) that were developed, standardized, and subsequently tested in a clinical pilot cohort of brain tumour patients, following a systematic method. Details of the steps taken during this process are outlined in the following sections.

## Materials and methods

**Fig. 1:**
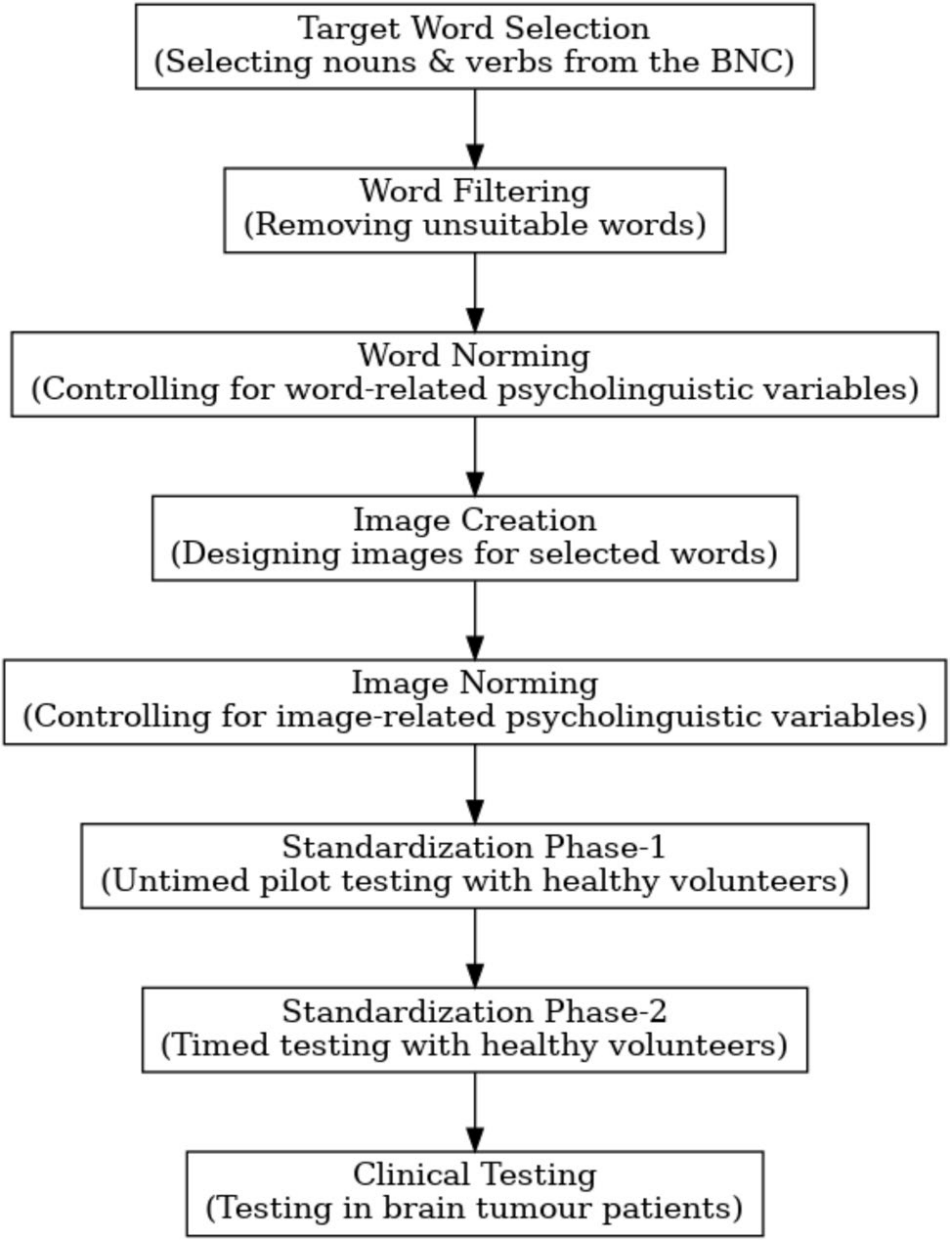
Workflow for BOATIM development and testing

To develop materials of BOATIM a step-wise procedure was followed: first, a list of target words was created using the spoken version of the British National Corpus (BNC) [5]. Selecting the target concepts from a representative spoken corpus rather than relying on translated materials ensured that the items were both linguistically and culturally appropriate to our target population. These words were then filtered to exclude any unsuitable items (see more details in the next section). Next, key concept- related psycholinguistic variables were controlled for by obtaining normative data from native British speakers. Corresponding images representing these controlled items were then sourced either from the existing picture databases or were created anew. Homogeneity across the image styles was ensured by controlling for image-related psycholinguistic variables. To standardise the stimuli, a two-step procedure (as reported in [30]) was followed: in step- 1 images were piloted with a small group of participants to exclude items that were not good representations of the target concept; in step- 2 the refined set of stimuli obtained from step- 1 were tested with a larger group to select items that were consistently named within 4000 ms, by at least 80% of the participants. These stages of development of BOATIM are illustrated in Fig. 1.

In the standardised data, we explored potential influences of subject-related (age and education) and psycholinguistic variables on task performance. To establish the robustness of the tasks, test–retest reliability was assessed. Finally, we evaluated the suitability of BOATIM as a measure for intraoperative language mapping and monitoring by administering the tests in a pilot cohort of brain tumour patients undergoing awake craniotomy.

### Word selection and norming

To select the candidate words, an initial list of 150 nouns and 150 verbs that we considered could be clearly and unambiguously depicted with an image was compiled from the spoken BNC [5] accessed via the BNC*web* tool [16]. All words were 1–2 syllable long and had varying degrees of frequency (ranging between 0.38–553.80 per million words for nouns and 0.19–763.17 per million words for verbs). The noun list consisted of only concrete nouns (representing several semantic categories, e.g., animals, furniture, vehicles), excluding the compounds (e.g., *toothbrush*) and the plural invariant forms (e.g., *scissors*) to trigger the single-word target response. The verb list comprised action verbs excluding the unaccusative and reflexive forms. The selected verbs were partially intransitive and had one argument, the agent (e.g., *to eat*), and partially pseudo-transitive (these carried a theme that does not need to be produced to form a grammatically correct sentence, e.g., *to read*). Furthermore, concepts with potentially negative connotations for the patients (e.g., *hospital*, *brain*, *to inject*) were avoided.

Given the subjective nature of some of the above criteria, some items for which the authors thought (i) visual depiction might hinder the unambiguous recognition of the target concept (e.g., concepts like *pillow*, *duvet*, *to repair*), (ii) various labels or longer than one-word answers could be generated (e.g., *pan*), or (iii) other criteria (e.g., verb transitivity/reflexivity) are not satisfied, were excluded. These comprised eight nouns and 20 verbs which reduced the initial item list to 142 nouns and 130 verbs in total.

Within lists, items were controlled for concept-related psycholinguistic variables. These included: frequency, imageability, concreteness, age-of-acquisition (AoA), length in phonemes, and verb transitivity and instrumentality. Corresponding values for frequency were extracted from the BNC. Verb transitivity and instrumentality was discussed between the authors until a consensus was reached. For imageability, concreteness, and AoA, subjective ratings from adult monolingual native British speakers (aged 18–70 years) with intact vision and no history of neurological and/or language-related illnesses and/or learning difficulties, were obtained. To do this, six different surveys (three per word class: one per variable) were conducted using the Qualtrics survey platform (https://www.qualtrics.com/). Participants (*N* = 50 per survey) were recruited via the Prolific interface (Prolific, Oxford; https://www.prolific.co). The detailed procedure of each survey, the rating scales used, and instructions per variable can be found in the supplementary materials (these are accessible at: https://osf.io/vzxar/?view_only=59a9bdeb78db40e0ab50fb27fce83556).

Results showed excellent inter-rater reliability for all variables (see Table 1 for the corresponding Cronbach’s alphas).

**Table 1:** Cronbach alpha per psycholinguistic variable across nouns and verbs

Variable	Cronbach’s α nouns	Cronbach’s α verbs	Number of raters
Imageability	0.98	0.99	50
Concreteness	0.99	0.98	50
AoA	0.98	0.98	50

Across the two word classes, there were no significant differences in frequency (*U* = 12,679, *p* = 0.399) and phoneme length (*t* = 1.68, *p* = 0.094). The values for imageability and concreteness were high for both nouns (imageability: *M* = 6.72, *SD* = 0.15; concreteness: *M* = 5.96, *SD* = 0.28) and verbs (imageability: *M* = 5.57, *SD* = 0.53; concreteness: *M* = 5.01, *SD* = 0.62). However, they could not be equated: imageability and concreteness of nouns was significantly higher than verbs (imageability: *U* = 18,435, *p* < 0.001; concreteness: *U* = 16,896, *p* < 0.001). This result is not surprising given the semantic nature of nouns being inherently more tangible and visually distinct than verbs which are primarily functional and motoric [4] [12].

AoA scores for both were lower (nouns: *M* = 2.10, *SD* = 0.60; verbs: *M* = 2.51, *SD* = 0.80), however, that of nouns were significantly lower than verbs (*U* = 6436, *p* < 0.001). This results supports the findings on early acquisition of nouns in English children (see for e.g., [4, 6], and [36]).

### Image design and norming

For nouns, the target images were mainly sourced from the MultiPic database [11]. For verbs, the image set of Cross Linguistic Lexical Tasks (CLT) [13] was used. Items for which no matches in the databases were found, or those that the authors considered were not representative of the British culture, were created anew by an illustrator. All images were colour illustrations with a black contour and portrayed against a white background. The overall graphic styles of the new set matched closely with the two databases used. To ensure further homogeneity, native British-English speakers (*N* = 50; aged 18–70 years) with intact colour vision and no history of neurological, and/or language-related illnesses and/or learning difficulties were asked to rate the images for visual complexity, that is, how complex the image is in terms of its surface details, and picture-name agreement (PNA), that is, how closely does the drawing match its name (see supplementary materials for survey methods including the instructions and rating scales used). Results showed excellent inter-rater reliability for both variables: visual complexity (*α* = 0.99, 50 raters – nouns; *α* = 0.99, 50 raters – verbs); PNA (*α* = 0.99, 50 raters – nouns; *α* = 0.99, 50 raters – verbs). As anticipated, owing to their dynamic nature, verbs had significantly higher visual complexity ratings (*U* = 4717.0, *p* < 0.001) but significantly lower PNA ratings (*U* = 12,246.0, *p* < 0.001) compared to nouns.

From the previously evaluated list, five nouns and two verbs were further excluded due to having visual complexity ratings above the median. This resulted in 137 noun and 128 verb stimuli to be included in the standardisation stage.

### Standardisation

As mentioned above, standardisation of the stimuli was done in two phases: phase- 1 (pilot phase) involved quality testing of the images in a small group under non-timed conditions. This allowed for exclusions of any unsuitable stimuli. Phase- 2 (standardisation phase) involved a large-scale validation of the remaining stimuli to select items that were consistently named within 4000 ms, by at least 80% of the participants.

## Pilot phase

### Participants

A total of *N* = 20 participants (12 female; *M* age = 39.4 years, age range = 24–62 years and *M* education = 16.9 years, education range = 12–24 years) were recruited in this phase. Inclusion criteria were: (1) right-handed, (2) adult monolingual speakers of British-English, (3) normal-to-corrected vision and hearing and no colour blindness, (3) no history of a neurological, cognitive, psychiatric, and/or a learning condition, and/or any drugs/alcohol abuse, and (5) have obtained above 24/30 score on the Standardised Mini Mental State Examination (MMSE) [8]. Participants were recruited primarily at the University of the West of England (UWE) Bristol, UK. All provided written consent and received a £10 voucher for their time.

### Procedure

Participants completed a self-paced oral naming experiment in a lab-based setting whereby they were shown the stimuli (269 images in total: 137 object and 128 action targets + 2 fillers per word type) via the PowerPoint presentation tool on a laptop computer. Images were displayed one-by-one against a white background, with a fixation cross preceding each presentation. The instruction was to name the depicted object or the action, using only a single word (i.e., the target noun or the verb associated with the depicted object/action; for actions, the continuous form e.g., *swimming* was requested to keep a similar structure across the responses). If two answers were given only the first one was considered. Responses were scored by the researcher during the experiment on a scoresheet and were also audio-recorded to verify the accuracy of the scoresheet afterwards. Object and action naming were completed as separate tasks (with a short 1–2-min interval), but their presentation was counterbalanced across the participants. Additionally, we created four different pseudo-randomized orders of the experiment. Participants were randomly assigned to one of the orders. Within each order, maximal semantic and phonological distances between the items (e.g., *monkey* and *rabbit* or *button* and *curtain* did not follow each other) were ensured [21].

Prior to the actual experiment, however, MMSE was administered as a screening test. Participants were only allowed to continue with the main experiment if they scored above 24/30 on the MMSE, a criterion that all successfully fulfilled. A practice task containing 10 items (five objects and five actions) was also administered ahead of the main experiment. The practice task was repeated until the participant demonstrated correct understanding of the task.

### Scoring and analysis

A response was considered correct if the pre-established linguistically controlled word was provided as a label. Responses that were anything other than the target word (e.g., synonyms, superordinate terms, multi-word expressions such as “fountain pen”, plurals, metonyms, and cohyponyms of the target word) were categorized as incorrect [29]. For each item, percentage of the correct answers was calculated. Items that were correctly named by at least 80% of the participants were considered to have passed a *naming agreement (NA) threshold* and only these items were considered for the subsequent standardisation phase [26] [31]. Furthermore, to account for response variability and the consistency of the dominant name given, we computed the H-statistic (using the formula in [32]). A cut-off score of an H-value of 0.85 was set. Items with a higher H-value were considered to have greater response variability and were therefore excluded [31].

Altogether 222 target images (125 objects and 97 actions) passed the 80% NA and the 0.85 H-statistic threshold in the pilot phase. A new test was created for the standardisation phase with these items.

### Standardisation phase

Here the selected material from the pilot phase was tested in a different group of healthy volunteers under the simulated DES conditions (i.e., with a 4000 ms response-time limit).

### Participants

A total of 54 volunteers took part in the standardisation phase (see participant demographics in Table 2). Since a ceiling level performance was expected the sample size could remain small [26]. Inclusion criteria for this group were identical to the pilot phase. This group was also recruited primarily at the UWE Bristol, UK. However, participants who took part in the pilot phase were excluded. Informed consent was obtained, and participants received a £10 voucher for their time.

**Table 2:** Demographics of participants in the standardisation phase

Gender	Mean age	Age range	Mean years of education	Education level range
40 female/14 males	37.2	19–65	17.2	11–23

A subset of the participants of this phase (*N* = 10, seven female, *M* = 32.6 years, age range: 19–38;) were retested a year after their initial participation to establish the test–retest reliability of BOATIM (see “Reliability” section below).

### Procedure

The overall procedure of this phase resembled the pilot study, i.e., participants first completed the MMSE, followed by a practice task (containing 10 items). Subsequently, they were assigned to one of the four pseudo-randomized versions of the experiment within which items were controlled for semantic and phonological distances. The presentation of object and action naming was counterbalanced across participants.

**Fig. 2:**
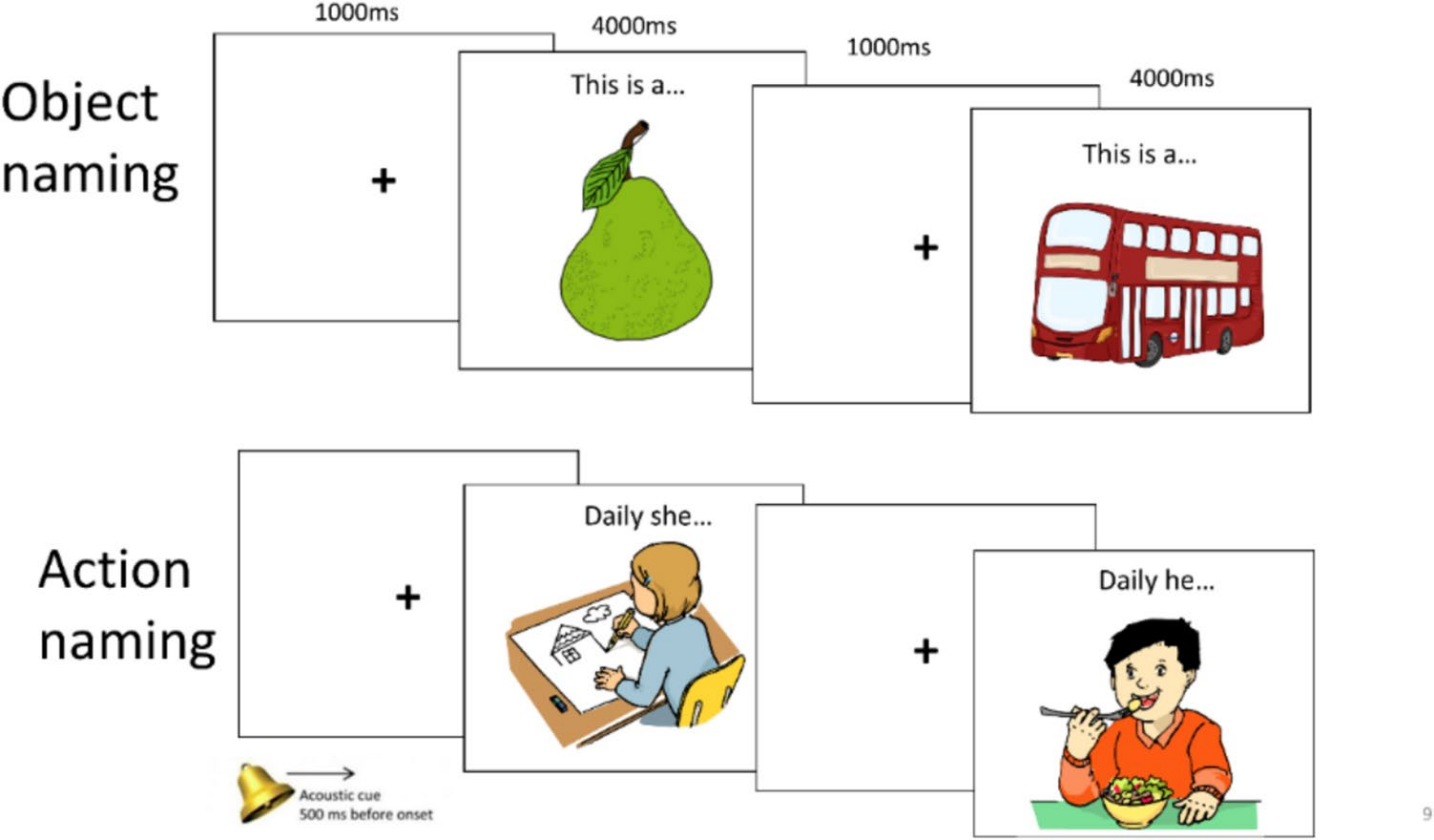
Example sequence of stimulus presentation

The key distinction in this phase was the duration of the stimulus presentation: to mimic the intraoperative DES settings, each image was displayed for a maximum of 4000 ms. Between images, a fixation cross appeared for 1000 ms which was accompanied by an acoustic cue 500 ms before the next image appeared. In addition, the task was performed in a short sentence context to trigger the production of lexical-grammatical processes. For this, a lead-in phrase “This is a/an…” (for objects) and “Daily he/she…” (for actions) was included above each image. Participants were required to read the phrase aloud and complete it with either a single noun corresponding to the depicted object, or a single verb (in its correct inflected form) appropriate for the depicted action, within the allocated 4000 ms time-limit. An example sequence of stimuli presentation is illustrated in Fig. 2.

The stimuli were presented via the Psychopy software [28] on a laptop computer. Participants responded to a total of 226 images (125 object + 97 action targets + 2 fillers per task type). Like the pilot phase, responses were marked on a scoresheet by the researcher and were also voice-recorded.

### Scoring and analysis

Data from nine participants was excluded due to technical issues. The responses of the remaining 45 participants were scored in the same manner as in the pilot phase. However, this time the 4000 ms time-limit was also taken into account: a response was considered correct only if the pre-established target word was provided as an answer within the allocated time.

Again, the NA and H-statistic was calculated for each item. Targets achieving the 80% NA threshold and that were below the 0.85 H-value were included in the item pool available for the development of the final set of tasks for clinical validation; the rest was excluded. A summary of the total number of items passing the NA and H-statistic threshold per task type in both phases is given in Table 3.

**Table 3:** Number of items passing the NA and H-statistic threshold over number of items tested in both phases

Task type	Pilot phase	Standardisation phase
Object naming	125/137	115/125
Action naming	97/128	86/97

In a series of follow-up analyses, we then explored participants’ performance differences across our two tasks, as well as the impact of several item- and subject-related variables on performance. We assessed the differences in exclusion rates between the object and the action naming task. For this, Barnard’s exact test was performed. We also compared naming accuracy (percentage of correct answers) using the Mann–Whitney *U* tests (as per non-normality of the data). Potential differences in performances due to subject-related factors, including age (two levels: above 35 median years: *N* = 21; and below 35 median years: *N* = 24) and education (two levels: higher – above 17 median education years: *N* = 26; or lower – below 17 median years of education: *N* = 19) were investigated by Kruskal–Wallis rank sum tests, as the data did not meet the assumption of normality.

Finally, we evaluated the influence of linguistic variables on naming accuracy using the Spearman rank correlations (due to violation of the normality assumption). To account for multiple comparisons, Bonferroni corrections were applied.

All analyses were performed using R Studio, Version 4.3.2.

### Results

201 target drawings (115 objects and 86 actions) passed the NA and the H-statistic threshold in the standardisation phase (see Table 3) and formed the basis of various statistical analyses reported here. Naming accuracy for object naming was significantly higher than for action naming (*W* = 1399.5, *p* = 0.002) with median accuracy scores of 98.26% and 95.35%, respectively (see Table 4). Consequently, a higher proportion of items were excluded in action naming (18.7%) compared to object naming (8.4%). This difference in exclusion rates was statistically significant (*S* = 2.49, *p* = 0.016, two-sided). Results are summarized in Table 5.

**Table 4:** Naming accuracy across tasks

Task	Median accuracy (%)	*W*	*p*
Object naming	98.26	1399.5	0.002
Action naming	95.35	-	-

**Table 5:** Item exclusion rates across tasks

Task	Exclusion rate (%)	*S*	*p*
Object naming	8.4	-	-
Action naming	18.7	2.49	0.016

For age, no statistically significant differences in naming accuracy were found across groups in either of the tasks (*X*^*2*^ = 0.87, *p* = 0.350 – object naming; *X*^*2*^ = 0.01, *p* = 0.090 – action naming). Education groups yielded similar results with no significant differences in performance across the two tasks (*X*^*2*^ = 0.14, *p* = 0.700 – object naming; *X*^*2*^ = 0.11, *p* = 0.737 – action naming). Results are reported in Table 6.

**Table 6:** Effect of age and education on naming accuracy across tasks

Variable	Task	*X*^*2*^	*p*
Age	Object naming	0.87	0.350
	Action naming	0.01	0.090
Education	Object naming	0.14	0.700
	Action naming	0.11	0.737

In terms of the relationship between the linguistic variables and naming accuracy, AoA correlated negatively with accuracy in both object naming (*r* = − 0.296, *p* = 0.006) and action naming (*r* = − 0.293, *p* = 0.030). Additionally, a positive effect of frequency was found across both tasks (*r* = 0.239, *p* = 0.050 – objects; *r* = 0.386, *p* = 0.001 – actions). No significant correlations were found for other variables. Results are reported in Table 7.

**Table 7:** Correlations (Spearman rank) between the linguistic variables and naming accuracies across tasks

	rho	*p* value after Bonferroni corrections
Object naming		
Frequency	0.239	0.050*
Imageability	0.111	1.000
AoA	− 0.296	0.006*
Concreteness	0.005	1.000
Number of phonemes	− 0.190	0.206
Animacy	0.022	1.000
Action naming		
Frequency	0.386	0.001*
Imageability	0.132	1.000
AoA	− 0.293	0.030*
Concreteness	0.269	0.060
Number of phonemes	0.035	1.000
Transitivity	− 0.071	1.000
Instrumentality	− 0.225	0.256

### Reliability

To explore test–retest reliability, BOATIM was administered twice in 10 randomly selected healthy participants from phase- 2 (since the performance on the items in this phase was at ceiling we considered the selected sample size to be sufficient). The duration between the two test points was approximately 12 months. Reliability was determined by Spearman correlations. For more robust assessment, single measure Interclass Correlation Coefficient (ICC) was computed with acceptability level set at 0.7 [23]. Excellent correlations were found between the scores of test-retests for both object naming (*r* = 0.92, *p* < 0.001) and action naming (*r* = 0.98, *p* < 0.001). ICC scores obtained were also excellent at 0.93 (*p* < 0.001, 95% CI: 0.88–0.96) for object and 0.99 (*p* < 0.001, 95% CI: 0.98–0.99) for action naming (see summary of the results in Table 8).

**Table 8:** Test–retest reliability across tasks

Task	rho	*p*	ICC (95% CI)	*p*
Object naming	0.92	*p* < 0.001	0.93 (0.88–0.96)	*p* < 0.001
Action naming	0.98	*p* < 0.001	0.99 (0.98–0.99)	*p* < 0.001

### Clinical testing

To demonstrate the clinical use of the BOATIM, a subset of the final items (*N* = 76 objects and *N* = 76 actions) was selected to be piloted in three adult and one paediatric patient presented with tumours in the language-eloquent regions. All patients were right-handed and native British speakers. The paediatric patient was also fluent in Polish. All were recruited at the Southmead Hospital, Bristol, UK. Pre- and post-operatively (at time-points 2–3 days and 2–6 weeks, respectively), BOATIM was administered together with tasks for other cognitive functions (e.g., attention, memory, reasoning). For intraoperative testing, an individually tailored testing battery as per the patient’s profile (e.g., pre-operative language ability, profession) and tumour characteristics (location) was prepared, following the recommendations of the DuLIP protocol [10]. The battery was practiced in the preoperative stage. Items that the patients performed incorrectly were removed. All patients provided written consent to be tested with the new stimuli and for their data to be included in this manuscript. Detailed case reports of patients and results of language mapping and monitoring using BOATIM follow.

### Case 1

**Fig. 3:**
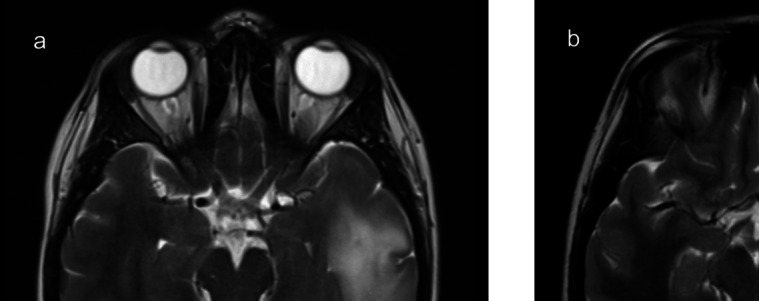
Case 1 – Preoperative MRI (axial view) demonstrating a left-temporal tumour with anterior structures highlighted (**a**). Post-operative MRI confirming complete resection (**b**)

A 27-year-old female developed generalized seizures with symptoms of memory disturbances. Magnetic resonance imaging (MRI) revealed a non-enhancing tumour in the left temporal lobe (Fig. 3). Preoperative language testing identified impairments in action word fluency and auditory naming. Intraoperatively, BOATIM object naming was administered during the mapping of superior temporal gyrus (STG), middle temporal gyrus (MTG), and inferior longitudinal fasciculus (ILF). Positive maps were obtained for STG. A complete resection of the tumour was achieved. Postoperatively, the patient exhibited persistent fluency difficulties, new reading and writing deficits lasting up to 6 weeks, and a superior visual quadrant field defect.

### Case 2

**Fig. 4:**
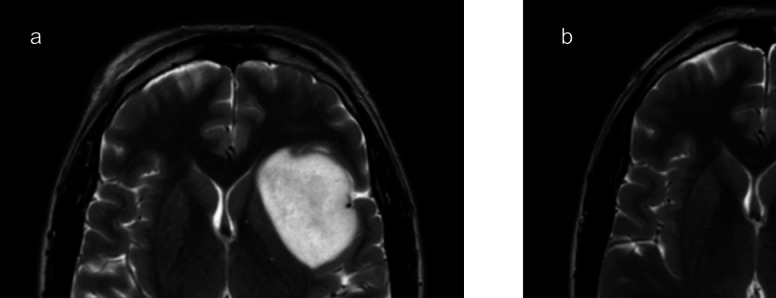
Case 2 – Preoperative MRI (axial view) demonstrated a left insular tumour (**a**). Post-operative MRI revealed a small volume of residual tumour in the posterior insula (**b**)

A 38-year-old male presented with seizures. A non-enhancing left insular tumour was detected on MRI (Fig. 4). Preoperative assessment showed low phonemic word fluency and difficulties in response inhibition, cognitive flexibility, and attention. No overt language deficits were observed. Intraoperatively, BOATIM action naming was used when stimulating inferior frontal gyrus (IFG) and middle frontal gyrus (MFG) while object naming mapped STG. Positive locations were found in the IFG and STG. Greater than 75% of the tumour was resected. At 6-week follow-up, no new deficits were present, and the patient was also able to return to work.

### Case 3

**Fig. 5:**
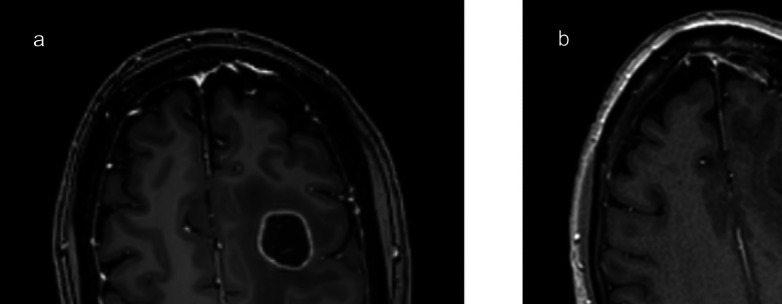
Case 3 – Preoperative MRI (axial view) demonstrated a metastasis in the left middle frontal gyrus (**a**). Post-operative MRI confirmed a complete resection (**b**)

Patient 3 was a 54-year-old female with a history of metastatic breast cancer. MRI showed an enhancing left frontal metastasis (Fig. 5). She initially presented with slurred speech, but no apparent deficits were found in the pre-operative assessment. Intraoperative functional mapping of MFG with BOATIM action naming resulted in positive maps. Tumour was fully excised. Post-operative follow-up confirmed preserved language and cognitive abilities.

### Case 4

**Fig. 6:**
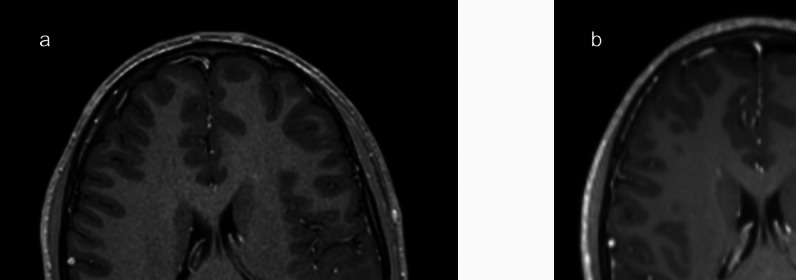
Case 4 – Pre-operative MRI (axial view) demonstrated an enhancing tumour in the left superior temporal gyrus (**a**). Post-operative imaging confirmed a complete resection (**b**)

Patient 4 was a 15-year-old English-Polish bilingual male who presented with focal seizures characterised by temporary aphasia, followed by a brief period of dysphasia. MRI showed an inhomogeneously enhancing tumour within the STG associated with surrounding oedema (Fig. 6). Preoperatively, low performance on language fluency and verbal reasoning task was observed. Intraoperative positive sites associated with BOATIM object naming included the STG. A follow-up evaluation after 14 days showed no language deficits [1].

## Discussion

This work provides the first tool designed specifically for intraoperative mapping in British English. Two picture-naming tasks were developed that were standardised in healthy volunteers and subsequently tested in brain tumour patients during their awake craniotomy. In preparing stimulus sets (representing objects and actions), we selected items from the spoken BNC to ensure their linguistic and cultural relevance to British speakers. Across sets, items were homogenous in terms of word frequency and length. Other psycholinguistic variables (i.e., imageability, concreteness and AoA) were accounted for by obtaining original ratings from the native speakers. Inter-rater reliability was excellent for all variables for the two word types. Ratings of imageability and concreteness were higher for both objects and actions but could not be equated given the semantic properties of objects [4]. As for the AoA, values for objects were lower compared to actions, which is in line with the reports of early acquisitions of nouns [6].

Amongst variables controlled for, AoA significantly influenced naming accuracy in both tasks where words learned earlier in life were associated with higher correctness scores. This result aligns with aphasiological research indicating that early word learning facilitates easier retrieval and access (which may be distinctly impaired as a result of brain damage including those caused by tumours) [25] [7]. A significant positive correlation of frequency was also found in both tasks, suggesting that higher frequency words are named more accurately. This result is also in line with previous evidence showing an effect of word frequency on naming accuracy [24]. In the light of these findings, we can say that BOATIM stimuli provide insights into factors impacting noun and verb retrieval.

BOATIM was standardised in a group of 45 healthy native volunteers. We did not find any clear effects of subject-related characteristics (i.e., age and education levels) on task performance. These results, in agreement with trends observed in other intraoperative batteries (see for e.g., [26]), may suggest that our tasks are not too challenging and may therefore be suitable for patients of diverse socio-demographics.

In comparison, task-related effects emerged: a significantly higher accuracy was found for object naming with a smaller number of items excluded than for action naming. This disparity does not suggest lower quality of the task or the individual items (as proven by the higher naming agreement of the final items) but can be attributed to the overall challenging nature of naming actions which requires describing a dynamic event/activity as opposed to naming a static object in isolation. Descriptions of events might also be influenced by individual interpretations. This was evident in more variable answers across participants leading to more errors in the task. Furthermore, the time-constraint of 4000 ms could have further added to the task difficulty resulting in lower accuracy. Consequently, action naming was overall a more complex task, both visually and linguistically. Existing batteries involving action naming report similar results (see for e.g., [26]).

In compliance with the standards of DES, our final stimuli only comprise items that can be named within 4000 ms. Excellent test–retest reliability in healthy participants was obtained for these items which indicates that BOATIM can provide a consistent and reliable evaluation of language skills.

More importantly, BOATIM was successfully administered during intraoperative functional mapping and eloquent regions were identified. This process also involved pre-surgery training of the items to ensure patient familiarity and item accuracy during mapping. Additionally, successful postoperative assessments were taken. The success of these applications can be attributed to linguistic specificity of the items that were shown to reliably elicit an expected response with minimal unexpected variants (e.g., synonyms). We take these and the above data to indicate that BOATIM is a more robust clinical alternative to translated or homemade tasks, capable of assessing language abilities and identification of speech-eloquent brain areas during neurosurgical testing.

Compared to limited item lists of extant batteries (e.g., *N* = 100 in VAN-POP and *N* = 40 in MULTIMAP), BOATIM provides a much larger stimulus set that can be tailored not only to create patient-specific versions of the tests but also to support broader experimental designs and cognitive studies.

BOATIM is developed specifically for neurosurgical language evaluation, especially for use during intraoperative language mapping. However, we do not disregard its potential to be used in other interventions, such as epilepsy surgery.

## Limitations and future directions

Our specific and targeted stimuli database, BOATIM, only allows for the evaluation of the language processes engaged by the naming of objects and actions in a short sentence context. Other essential aspects of speech (e.g., complex syntactic processes) are not covered in the same range. Future studies could use the structured method of test development adapted here as a guide to design more comprehensive batteries tapping into more complex functions (see [27] for a detailed discussion on this). Stimuli should also be validated in a larger cohort of patients to better understand individual differences and to assure the state-of-the-art testing procedure is robust to detecting subtle language errors induced by DES. Expanding the pool of participants will also allow for comparisons of potential influences of psycholinguistic variables on task performance (which in current study was limited to healthy volunteers only due to small sample size of the clinical data). Additionally, only native British speakers were tested. Administering BOATIM in other dialects of English might be of great value towards establishing its generalizability and obtaining multicentre comparative data.

We did not find any subject-related differences in the task performance (that were only analysed in the healthy participant data due to a larger sample). However, we acknowledge that our participant pool was recruited exclusively from a university setting, including students, academic staff and professional services members. Specifically, in terms of education, while the sample covered a broad range of education levels (11–23 years), participants may have had greater exposure to academic environments, potentially influencing language processing efficiency to a certain degree. Future studies should continue to include individuals from diverse educational and socioeconomic backgrounds, such as those in non-academic professions, vocational training, and individuals with fewer years of formal education to further validate our findings. Moreover, our sample was not balanced for gender with more female participants than male. Controlling for these features in future analyses is likely to confirm BOATIM’s applicability in an even more balanced population.

Finally, an excellent test–retest reliability was obtained for BOATIM items across both tasks. However, our sample size – due to being restricted by participants availability – was relatively small. Although we considered this sample sufficient given the ceiling-level performance observed in the Standardization phase, statistical literature suggests that a minimum of 20 participants is more ideal for reliability estimation (see for e.g., [22]). Thus, our findings should be interpreted as preliminary, and future studies should aim to validate reliability with a larger sample to ensure the robustness of the estimates.

## Supplementary Information

Below is the link to the electronic supplementary material.

Supplementary file1 (XLSX 58.5 KB)

## Data Availability

The list of stimuli (in alphabetical order) corresponding to the two tests, together with information on their norming data, are accessible on this OSF link: https://osf.io/vzxar/?view_only=59a9bdeb78db40e0ab50fb27fce83556.

## Abbreviations

*AoA*: Age-of-acquisition
*BNC*: British National Corpus
*DES*: Direct electrical stimulation
*DuLIP*: The Dutch linguistic intraoperative protocol
*VAN-POP*: The verb and noun test for peri-operative testing
*MULTIMAP*: Multilingual picture naming test for mapping eloquent areas during awake surgeries
*MultiPic*: Multilingual picture naming database
*STG*: Superior temporal gyrus
*MTG*: Middle temporal gyrus
*ILF*: Inferior longitudinal fasciculus
*IFG*: Inferior frontal gyrus
*MFG*: Middle frontal gyrus
